# Optimal Proteinuria Target for Renoprotection in Patients with IgA Nephropathy

**DOI:** 10.1371/journal.pone.0101935

**Published:** 2014-07-08

**Authors:** Ki Heon Nam, Jeong Hae Kie, Mi Jung Lee, Tae-Ik Chang, Ea Wha Kang, Dong Wook Kim, Beom Jin Lim, Jung Tak Park, Young Eun Kwon, Yung Ly Kim, Kyoung Sook Park, Seong Yeong An, Hyung Jung Oh, Tae-Hyun Yoo, Shin-Wook Kang, Kyu Hun Choi, Hyeon Joo Jeong, Dae-Suk Han, Seung Hyeok Han

**Affiliations:** 1 Division of Nephrology, Department of Internal Medicine, College of Medicine, Yonsei University, Seoul, Republic of Korea; 2 Department of Pathology, NHIS Ilsan Hospital, Goyang-shi, Gyeonggi-do, Republic of Korea; 3 Department of Internal Medicine, NHIS Ilsan Hospital, Goyang-shi, Gyeonggi-do, Republic of Korea; 4 Biostatistics Collaboration Unit, College of Medicine, Yonsei University, Seoul, Republic of Korea; 5 Department of Pathology, College of Medicine, Yonsei University, Seoul, Republic of Korea; 6 Severance Biomedical Science Institute, Brain Korea 21, Yonsei University, Seoul, Republic of Korea; National Center for Scientific Research Demokritos, Greece

## Abstract

**Background:**

Proteinuria is a target for renoprotection in kidney diseases. However, optimal level of proteinuria reduction in IgA nephropathy (IgAN) is unknown.

**Methods:**

We conducted a retrospective observational study in 500 patients with biopsy-proven IgAN. Time-averaged proteinuria (TA-P) was calculated as the mean of every 6 month period of measurements of spot urine protein-to-creatinine ratio. The study endpoints were a 50% decline in estimated glomerular filtration rate (eGFR), onset of end-stage renal disease (ESRD), and slope of eGFR.

**Results:**

During a median follow-up duration of 65 (12–154) months, a 50% decline in eGFR occurred in 1 (0.8%) patient with TA-P of <0.3 g/g compared to 6 (2.7%) patients with TA-P of 0.3–0.99 g/g (hazard ratio, 2.82; *P* = 0.35). Risk of reaching a 50% decline in eGFR markedly increased in patients with TA-P of 1.0–2.99 g/g (*P* = 0.002) and those with TA-P≥3.0 g/g (*P*<0.001). ESRD did not occur in patients with TA-P<1.0 g/g compared to 26 (20.0%) and 8 (57.1%) patients with TA-P of 1.0–2.99 and ≥3.0 g/g, respectively. Kidney function of these two groups deteriorated faster than those with TA-P<1.0 g/g (*P*<0.001). However, patients with TA-P of 0.3–0.99 g/g had a greater decline of eGFR than patients with TA-P<0.3 g/g (−0.41±1.68 vs. −0.73±2.82 ml/min/1.73 m^2^/year, *P* = 0.03).

**Conclusion:**

In this study, patients with TA-P<1.0 g/g show favorable outcomes. However, given the faster eGFR decline in patients with TA-P of 0.3–0.99 g/g than in patients with TA-P<0.3 g/g, the ultimate optimal goal of proteinuria reduction can be lowered in the management of IgAN.

## Introduction

IgA nephropathy (IgAN) is slowly progressive and 20–30% of patients with IgAN will require renal replacement therapy within 20–25 years after disease onset [1], [2]. Because it is not a totally benign condition, a number of studies have identified risk factors associated with progression of IgAN. Among these, proteinuria is considered a strong predictor of adverse renal outcome [3]–[13]. In fact, proteinuria is significantly related to clinical and pathological factors that affect the future outcome in many glomerular diseases; thus is a therapeutic target for kidney protection. Treatment options for achieving reducing proteinuria differ according to the type and severity of disease. As aforementioned, unlike idiopathic nephrotic syndrome or crescentic glomerulonephritis showing abrupt onset or a rapidly progressive decline in renal function, IgAN is characterized by slowly progressive nature without symptoms over time. Therefore, it is difficult to determine when to treat or who should be treated. In addition, therapeutic options are limited and the role of immunosuppressive drugs has not yet been clearly defined. Nevertheless, reducing proteinuria is of paramount importance to improve prognosis in these patients. However, the optimal target for proteinuria reduction to attenuate progression of kidney disease is currently unknown. Interestingly, the target may differ depending on the type of proteinuric glomerular disease [14]. Moreover, although a favorable outcome is more likely for patients with IgAN that have proteinuria <1 g/day throughout the disease course, there is controversy on whether further reduction of proteinuria below this level will provide additional benefit [11], [12]. The purpose of this study was, therefore, to identify the optimal target for proteinuria reduction for renoprotection in patients with IgAN. To this end, we used time-averaged proteinuria (TA-P) and classified patients into four groups according to TA-P levels. In particular, we aimed to investigate whether reducing proteinuria below the level that the current guideline suggests may improve renal outcome.

## Materials and Methods

### Patient Selection

**Figure 1:**
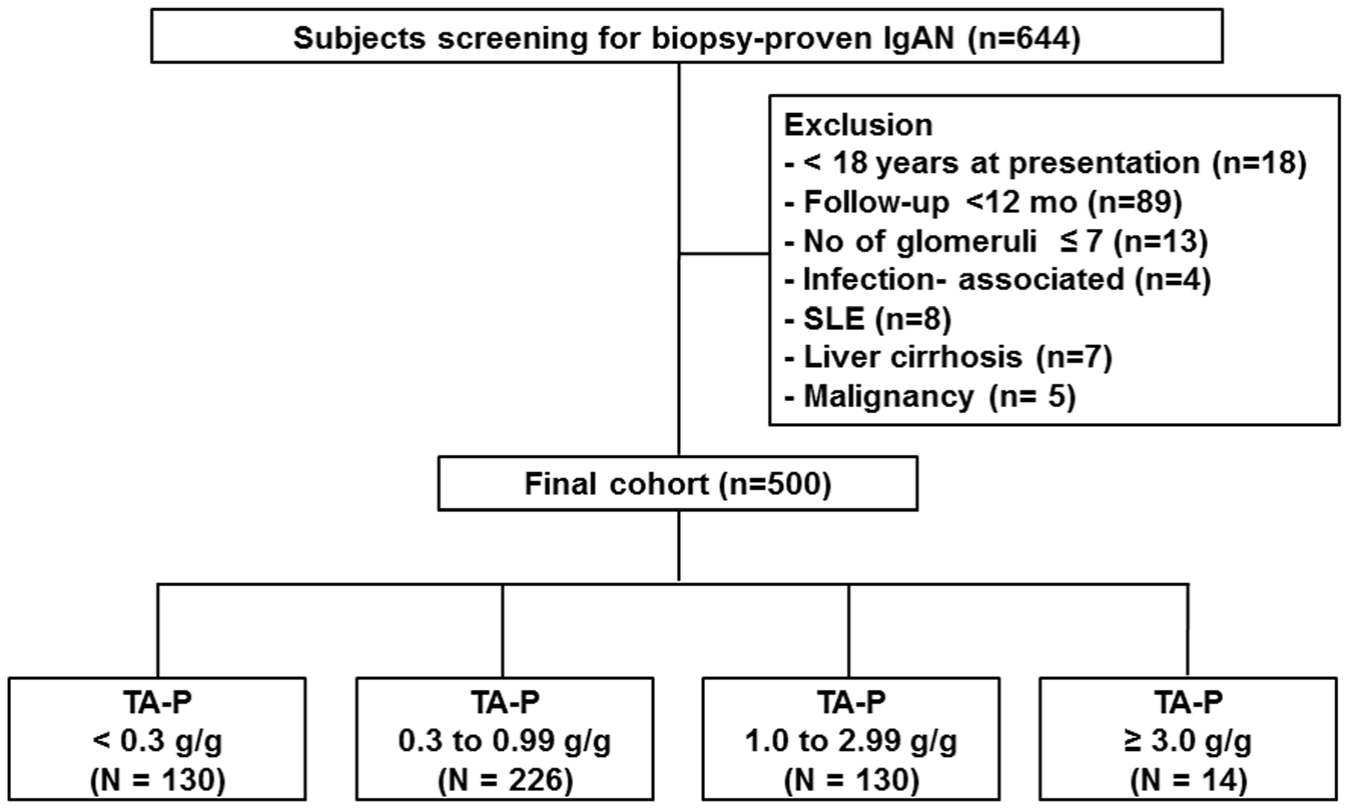
Flow chart of participants. Abbreviations: IgAN, Immunoglobulin A nephropathy; SLE, systemic lupus erythematosus; TA-P, time-averaged proteinuria.

A flow chart of participants is shown in Figure 1. A total of 644 patients were pathologically diagnosed with IgAN in Yonsei University Severance Hospital and National Health Insurance Services Ilsan Hospital between 2002 and 2010. All patients had definite pathologic findings with predominant mesangial deposition of IgA with at least 1+ on immunofluorescent staining and electron-dense deposits within the mesangium detected by electron microscopy. Patients with Henoch-Schonlein purpura nephritis were considered ineligible. Exclusion criteria were as follows: aged <18 years (n = 18); follow-up duration <12 months (n = 89); inadequate biopsy sample containing ≤7 glomeruli (n = 13); secondary causes of mesangial IgA deposition, such as IgA-dominant acute post-infectious glomerulonephritis (n = 4); systemic lupus erythematosus (n = 8); liver cirrhosis (n = 7); or malignancy (n = 5). Therefore, a total of 500 patients were analyzed in this study. We carried out the study in accordance with the Declaration of Helsinki, and the study was approved by the Institutional Review Board (IRB) of Yonsei University Health System Clinical Trial Center. Since the current study was a retrospective study and the study subjects were de-identified, the IRB waived the need for written consent from the patients.

### Data collection

Demographic and clinical data were collected at the time of renal biopsy. These included gender, age, systolic and diastolic blood pressure (BP), comorbidities, date of renal biopsy, pathologic findings, and laboratory parameters. Estimated glomerular filtration rate (eGFR) was calculated using the CKD-EPI equation [15]. During follow-up, proteinuria was assessed by spot urine protein-creatinine ratio (UPCR) because measurement of 24-h protein excretion was not feasible at all visits. Using these data, we calculated TA-P as an average of the means of every 6 month period of proteinuria measurements [11]. TA-BP was also determined in the same way. In addition, medications including anti-hypertensives, renin-angiotensin system (RAS) blockers, and immunosuppressants were recorded. In this study, we presented pathologic findings using the Oxford classification criteria [16], [17].

We also determined the absolute renal risk (ARR) score at the time of diagnosis as suggested by Berthoux et al [4]. ARR was scored from 0 to 3 depending on the number of risk factors; (1) the presence of hypertension, (2) proteinuria ≥1.0 g/g, and (3) renal pathologic lesion. For renal lesion, instead of global optical score used in the original study by Berthoux et al, MEST score was determined using the sum of each lesion by the Oxford classification. We performed ROC analyses and found that MEST score ≥2.0 had the highest AUC value (0.744), thus was considered as a score of 1 for pathologic lesion. We evaluated the effect of proteinuria reduction according to each score of ARR.

### Study endpoints

Study endpoints were a 50% decline in eGFR, the onset of end-stage renal disease (ESRD), and slope of eGFR. A 50% decline in eGFR was defined as a sustained decrease in eGFR>50% for at least three consecutive measurements. The first of these consecutive measurements was retrospectively designated to be the 50% decline in eGFR endpoint. ESRD was defined as initiation of dialysis or receipt of a transplant.

### Statistical Analyses

All variables with normal distribution were expressed as mean ± standard deviation. Comparisons were made by Student's t-tests or one-way ANOVA for continuous variables and by the Chi-square test for categorical variables as required. If data did not have a normal distribution, they were expressed as median and interquartile range and were compared using the Mann–Whitney test or the Kruskal–Wallis test. The cumulative renal survival rates were estimated by the Kaplan-Meier method and differences between survival curves were compared with the log-rank test. Renal survival time was defined as the interval between the time of biopsy and last follow-up. We determined the slope of the eGFR decline using multiple linear regression analyses. A Cox proportional hazards model was used to identify independent variables affecting renal survival. Variables with a p-value<0.10 in the univariate analysis were entered into the multivariate analysis. The results were expressed as a hazard ratio (HR) and 95% confidence interval (CI). All *P*-values were two-tailed and values <0.05 were considered statistically significant. Data analysis was performed using SPSS version 18 software for Windows (SPSS, Chicago, IL).

## Results

### Patient characteristics according to TA-P

Patient characteristics of the study population are shown in Table 1. The mean age at the time of renal biopsy was 37.0±12.0 years and 215 patients (43%) were male. 122 (24.4%) patients were hypertensive before renal biopsy. The median 24-h protein excretion was 0.5 (0.1–1.5) g/day and mean eGFR was 80.2±23.4 ml/min/1.73 m^2^. When pathologic findings were analyzed using the Oxford classification system, there were 205 (41.0%), 48 (9.6%), 209 (41.8%), 53 (10.6%) and 36 (7.2%) patients with M1, E1, S1, T1, and T2 lesions, respectively.

**Table 1:** Patient characteristics according to time-averaged proteinuria.

		Time-averaged proteinuria (g/g)				
Variables	Total	<0.3	0.3–0.99	1.0–2.99	≥3.0	*P*-for trend
Number	500	130 (26.0%)	226 (45.2%)	130 (26.0%)	14 (2.8%)	
Male (n, %)	215 (43%)	76 (58.5%)	85 (37.6%)	50 (38.5%)	10 (28.6%)	0.01*
Age (years)	37.1±12.0	34.2±12.3	38.0±11.6	38.4±12.0	35.6±11.4	0.08
Hypertension (n, %)	122 (24.4%)	21 (16.2%)	59 (26.1%)	38 (29.2%)	4 (28.6%)	0.06*
Hypertension at last follow-up (n, %)	178 (35.6%)	33 (25.4%)	77 (34.1%)	61 (46.9%)	7 (50.0%)	0.002*
TA-SBP (mmHg)	121.5±8.5	120.3±8.0	120.7±8. 5	123.6±8.6	126.4±8.9	<0.001
TA-DBP (mmHg)	75.5±6.4	74.1±5.9	75.2±6.5	77.0±6.3	78.1±6.0	<0.001
TA-MAP (mmHg)	90.8±6.6	89.5±6.0	90.4±6.8	92.5±6.6	94.2±5.9	<0.001
BUN (mg/dl)	15.0±5.7	13.2±3.9	14.9±5.1	16.8±6.9	17.9±9.5	<0.001
S-Cr (mg/dl)	1.0±0.4	1.0±0.3	1.0±0.3	1.1±0.5	1.2±0.6	<0.001
eGFR (ml/min per 1.73 m^2^)	87.3±28.5	89.0±25.0	89.8±26.3	81.8±33.8	83.4±35.5	0.04
eGFR stage (ml/min per 1.73 m^2^)						
≥90 (n, %)	241	63 (48.5%)	119 (52.7%)	53 (40.8%)	6 (42.9%)	0.18*
60–89 (n, %)	162	51 (39.2%)	70 (31.0%)	38 (29.2%)	3 (21.4%)	0.23*
30–60 (n, %)	84	15 (11.5%)	35 (15.5%)	30 (23.1%)	4 (28.6%)	0.05*
15–30 (n, %)	13	1 (0.8%)	2 (0.9%)	9 (6.9%)	1 (7.1%)	0.002*
Serum albumin (g/dl)	4.0±0.6	4.3±0.5	4.0±0.5	3.8±0.6	3.0±0.8	<0.001
Total cholesterol (mg/dl)	190.0±50.0	175.7±46.0	186.1±36.2	203.3±61.7	235.2.9±69.2	<0.001
Triglyceride (mg/dl)†	110.0 (80.0–161.0)	98.0 (75.0–137.8)	104.5 (74.5–160.0)	127.5 (91.0–201.3)	130.0 (99.5–158.5)	0.001
Hb (g/dl)	13.1±2.0	13.7±1.4	13.2±1.9	13.0±2.2	10.2±3.0	<0.001
UPCR (g/g)†	0.9 (0.4–1.8)	0.3 (0.1–0.6)	0.9 (0.5–1.4)	1.8 (1.1–3.0)	4.7 (2.9–6.1)	<0.001
Proteinuria (g/24 h)†	0.5 (0.1–1.5)	0.2 (0.1–0.4)	0.6 (0.1–1.2)	1.3 (0.8–2.9)	2.9 (1.1–4.4)	<0.001
Treatment (n, %)						
RAS blockers	390 (78.0%)	66 (50.8%)	188 (83.2%)	123 (94.6%)	13 (92.9%)	<0.001*
Corticosteroids	55 (11.0%)	6 (4.6%)	25 (11.1%)	17 (13.1%)	7 (50.0%)	<0.001*
Fish oil	20 (4.0%)	5 (3.8%)	10 (4.4%)	3 (2.3%)	2 (14.3%)	0.176*
Oxford-MEST (n, %)						
M1	205 (41.0%)	31 (23.8%)	91 (40.3%)	75 (57.7%)	8 (57.1%)	<0.001*
E1	48 (9.6%)	5 (3.8%)	22 (9.7%)	18 (13.8%)	3 (21.4%)	0.019*
S1	209 (41.8%)	29 (22.3%)	115 (50.9%)	61 (46.9%)	4 (28.6%)	<0.001*
T1	53 (10.6%)	3 (2.3%)	19 (8.4%)	27 (20.8%)	4 (28.6%)	<0.001*
T2	36 (7.2%)	1 (0.8%)	5 (1.0%)	26 (20.0%)	4 (28.6%)	<0.001*
MEST score	1.2±1.2	0.5±0.7	1.1±1.0	1.8±1.3	1.9±1.4	<0.001
ARR score	1.0±1.0	0.4±0.7	1.0±0.9	1.6±0.9	1.8±0.8	<0.001
0 (n, %)	184 (36.8%)	92 (50.0%)	74 (40.2%)	17 (9.2%)	1 (0.5%)	<0.001*
1 (n, %)	149 (29.8%)	25 (16.8%)	85 (57.0%)	36 (24.2%)	3 (2.0%)	<0.001*
2 (n, %)	132 (26.4%)	13 (9.8%)	53 (40.2%)	58 (43.9%)	8 (6.1%)	<0.001*
3 (n, %)	35 (7.0%)	0 (0.0%)	14 (6.2%)	19 (14.2%)	2 (14.3%)	<0.001*

All data are expressed as mean ± SD or

† median (and interquartile range).

*Statistical analyses were done using chi-square tests.

Abbreviations: TA-SBP, time-averaged systolic blood pressure; TA-DBP, time-averaged diastolic blood pressure; TA-MAP, time-averaged mean arterial pressure; BUN, blood urea nitrogen; S-Cr, serum creatinine; eGFR, estimated glomerular filtration rate; Hb, hemoglobin; UPCR, urine protein-to-creatinine ratio; RAS, renin-angiotensin system; ARR, absolute renal risk.

Patients were categorized into four groups according to TA-P (Table 1). 130 (26.0%), 226 (45.2%), 130 (26.0%), and 14 (2.8%) patients had a TA-P of <0.3, 0.3–0.99, 1.0–2.99, and ≥3.0 g/g, respectively. There were no significant differences in age, sex, or comorbidities, between the four groups. However, TA-BP, serum concentrations of total cholesterol and triglycerides, 24-h urine protein excretion, urine UPCR, MEST score, and ARR score were significantly higher in patients with higher TA-P (*P* for trend <0.001). eGFR (*P* for trend = 0.04) and serum albumin levels (*P* for trend <0.001) were lower in patients with higher TA-P. During follow-up, patients with TA-P<0.3 g/g were less treated with RAS blockers than other groups. In addition, corticosteroids were most commonly prescribed to patients with TA-P≥3.0 g/g.

### Changes in eGFR, CKD stage, proteinuria, and BP during follow-up

Median follow-up duration in the cohort was 65 (12–154) months. Data for eGFR, CKD stage, proteinuria, and BP at the time of diagnosis and last follow-up are presented in Table 2. eGFR decreased from 87.3±28.5 to 76.9±32.8 ml/min per 1.73 m^2^. Accordingly, there were more patients with higher CKD stages at last follow-up. Proteinuria decreased from 0.9 (0.4–1.8) to 0.4 (0.1–1.0) g/g during this period. At the time of diagnosis, 122 (24.4%) patients had hypertension. Baseline systolic and diastolic blood pressures of these patients were 136.7±18.2 and 84.0±11.9 mmHg, respectively. During the follow-up period, 56 (11.2%) patients newly developed hypertension. Of 110 hypertensive patients with TA-P<1.0 g/g, 61 (55.5%) achieved BP≤130/80 mmHg. In addition, 12 (17.6%) patients of 68 hypertensive patients with TA-P≥1.0 g/g achieved BP≤125/75 mmHg.

**Table 2:** Changes in eGFR, CKD stage, proteinuria, and blood pressure.

	Diagnosis	Last follow up	*P*-value
eGFR (ml/min per 1.73 m^2^)	87.3±28.5	76.9±32.8	<0.001
CKD stage (n, %)			
≥90	241 (48.2%)	202 (40.4%)	
60–89	162 (32.4%)	158 (31.6%)	
30–59	84 (16.8%)	84 (16.8%)	<0.001
15–29	13 (2.6%)	19 (3.8%)	
<15	0 (0.0%)	37 (7.4%)	
Proteinuria (g/g) (n, %)	0.9 (0.4–1.8)	0.4 (0.1–1.0)	<0.001
<0.3	130 (26.0%)	229 (45.8%)	
0.3–0.99	226 (45.2%)	147 (29.4%)	<0.001
1.0–2.99	130 (26.0%)	88 (17.6%)	
≥3.0	14 (2.8%)	36 (7.2%)	
Hypertension (yes; n, %)	122 (24.4%)	178 (35.6%)	
SBP (mmHg)	136.7±18.2	130.1±14.5	0.001
DBP (mmHg)	84.0±11.9	81.8±10.9	0.09
Hypertension developed during follow-up (n, %)	-	56 (11.2%)	
Hypertension (no; n, %)	378 (75.6%)	322 (64.4%)	
SBP (mmHg)	122.7±12.9	117.6±9.8	<0.001
DBP (mmHg)	76.5±10.2	73.4±7.7	<0.001
Among patients with hypertension			
TA-P<1.0 g/g (n, %)	-	110 (61.8%)	
TA-P≥1.0 g/g (n, %)	-	68 (38.2%)	
BP controlled (n, %)			
≤130/80 mmHg	-	87 (48.9%)	
≤130/80 mmHg (TA-P<1.0 g/g)	-	61 (55.5%)	
≤125/75 mmHg (TA-P≥1.0 g/g)	-	12 (17.6%)	

Abbreviations: eGFR, estimated glomerular filtration rate; CKD, chronic kidney disease; SBP, systolic blood pressure; DBP, diastolic blood pressure; TA-P, time-averaged proteinuria.

### Renal outcomes according to TA-P

As shown in Table 3, 60 (12.0%) patients reached a 50% decline in eGFR during follow-up. In addition, 34 (6.8%) patients developed ESRD. There was no patient who progressed to ESRD before reaching a 50% decline in eGFR. In addition, no death occurred before ESRD developed.

**Table 3:** Clinical outcomes according to time-averaged proteinuria.

			Time-averaged proteinuria (g/g)								
			<0.3		0.3–0.99		1.0–2.99		≥3.0		
	All	/100 Patient-years	N (%)	/100 Patient-years	N (%)	/100 Patient-years	N (%)	/100 Patient-years	N (%)	/100 Patient-years	*P*-value
eGFR decline >50%	60 (12.0%)	1.84	1 (0.8%)	0.13	6 (2.7%)	0.47	44 (33.8%)	6.34	9 (64.3%)	22.36	<0.001
ESRD	34 (6.8%)	1.23	0 (0%)	0	0 (0%)	0	26 (20.0%)	3.75	8 (57.1%)	19.9	<0.001

Abbreviations: eGFR, estimated glomerular filtration rate; ESRD, end-stage renal disease.

**Figure 2:**
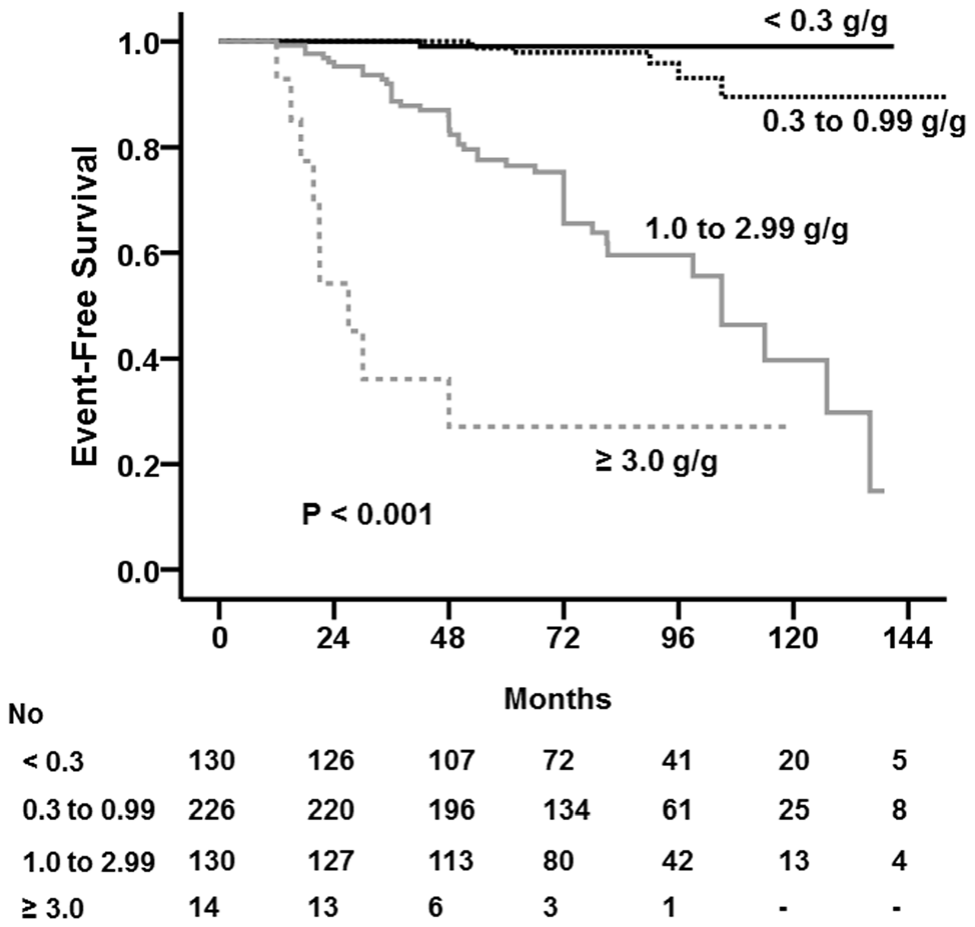
The Kaplan–Meier renal survival curve of patients with IgAN according to time averaged proteinuria (TA-P). Renal survival rates were lower as patients had greater amount of TA-P, particularly from TA-P>1.0 g/g. There was no significant difference in renal survival rate between patients with TA-P<0.3 g/g and TA-P of 0.3–0.99 g/g.

We further analyzed renal outcomes according to TA-P. A 50% decline in eGFR was most commonly reached in patients with TA-P≥3.0 g/g (64.3%), followed by those with TA-P of 1.0–2.99 g/g (33.8%). There was no significant difference in the development of a 50% decline in eGFR between patients with TA-P<0.3 g/g (0.8%) and those with TA-P of 0.3–0.99 g/g (2.7%) (*P* = 0.22). ESRD occurred in 8 (57.1%) and 26 (20.0%) patients with TA-P≥3.0 g/g and 1.0–2.99 g/g, respectively, whereas it did not occur in any patients with TA-P<1.0 g/g. A Kaplan–Meier curve also showed that renal survival rates were lower as patients had greater TA-P (Figure 2), particularly from TA-P>1.0 g/g. There was no significant difference in renal survival rate between patients with TA-P<0.3 g/g and TA-P of 0.3–0.99 g/g. Their 10-year survival rates were excellent, which were 99.0% and 97.9%, respectively (*P* = 0.171).

### Multivariable Cox models for renal outcome of a 50% decline in eGFR

To determine HRs according to TA-P groups, we constructed multivariable Cox models where the four TA-P groups were entered after adjustment for clinical parameters and pathologic findings (Table 4). The risk of reaching a 50% decline in eGFR did not differ between patients with TA-P<0.3 g/g and those with TA-P of 0.3–0.99 g/g in model 1 adjusted for age, mean arterial pressure, the presence of hypertension, and eGFR (HR, 3.45; 95% CI, 0.41 to 28.80; *P* = 0.25). The HR was not significantly altered by addition of pathologic findings to model 1 (HR, 2.93; 95% CI, 0.35 to 24.98; *P* = 0.33) (Table 4, Model 2). Furthermore, the model that was fully adjusted for the use of RAS blockers and corticosteroids showed no significant increase in the risk of reaching a 50% decline in eGFR in patients with TA-P of 0.3–0.99 g/g, versus those with TA-P<0.3 g/g (HR, 2.82; 95% CI, 0.32 to 24.72; *P* = 0.35) (Table 4, Model 3). When ARR score was entered in the model instead of presence of hypertension, and MEST score, we produced the same results (HR, 2.60; 95% CI, 0.30 to 22.91; *P* = 0.388; data not shown). Of note, risk of progression was markedly increased in patients with TA-P of 1.0–2.99 g/g and highest in patients with TA-P>3.0 g/g. Such increased risks in these groups were consistently noted in all three models.

**Table 4:** Multivariable Cox regression models for renal outcome of decline in eGFR>50%.

	Model 1		Model 2		Model 3	
Variables	HR (95% CI)	*P*-value	HR (95% CI)	*P*-value	HR (95% CI)	*P*-value
Age (1 year)	0.97 (0.95 to 1.00)	0.030	0.98 (0.95 to 1.01)	0.90	0.98 (0.95 to 1.03)	0.083
eGFR (1 ml/min/1.73 m^2^)	0.97 (0.96 to 0.98)	<0.001	0.98 (0.97 to 0.99)	<0.001	0.98 (0.97 to 0.99)	<0.001
TA_MAP (1 mmHg)	1.03 (0.98 to 1.08)	0.22	1.04 (0.99 to 1.09)	0.13	1.04 (0.99 to 1.09)	0.17
Hypertension (vs. no)	1.23 (0.69 to 2.20)	0.48	0.95 (0.52 to 1.78)	0.88	0.97 (0.52 to 1.80)	0.97
TA-P (g/g)						
<0.3	reference		reference		reference	
0.3 to 0.99	3.45 (0.41 to 28.80)	0.25	2.93 (0.35 to 24.98)	0.33	2.82 (0.32 to 24.72)	0.35
1.0 to 2.99	47.28 (6.38 to 350.15)	<0.001	26.59 (3.48 to 203.34)	0.002	25.0 (3.17 to 197.0)	0.002
≥3.0	406.35 (49.30 to 3349.18)	<0.001	288.86 (34.03 to 2451.86)	<0.001	244.07 (27.09 to 2198.88)	<0.001
Pathologic findings						
M1 (vs. M0)	-	-	1.30 (0.64 to 2.66)	0.47	1.33 (0.65 to 2.75)	0.44
E1 (vs. E0)	-	-	0.97 (0.43 to 2.17)	0.94	0.94 (0.42 to 2.11)	0.87
S1 (vs. S0)	-	-	1.05 (0.59 to 1.88)	0.86	1.01 (0.56 to 1.82)	0.97
T1 (vs. T0)	-	-	2.78 (1.29 to 5.99)	0.009	2.81 (1.29 to 6.11)	0.009
T2 (vs. T0)	-	-	4.27 (1.80 to 10.17)	0.001	4.35 (1.81 to 10.42)	0.001
Treatment						
RAS blockers (vs. no)	-	-	-	-	1.11 (0.30 to 4.06)	0.88
Corticosteroids (vs. no)	-	-	-	-	1.37 (0.65 to 2.88)	0.41

Abbreviations: HR, hazard ratio; eGFR, estimated glomerular filtration rate; TA-MAP, time-averaged mean arterial pressure; TA-P, time-averaged proteinuria; RAS, renin-angiotensin system.

### Proteinuria reduction and renal outcome

**Figure 3:**
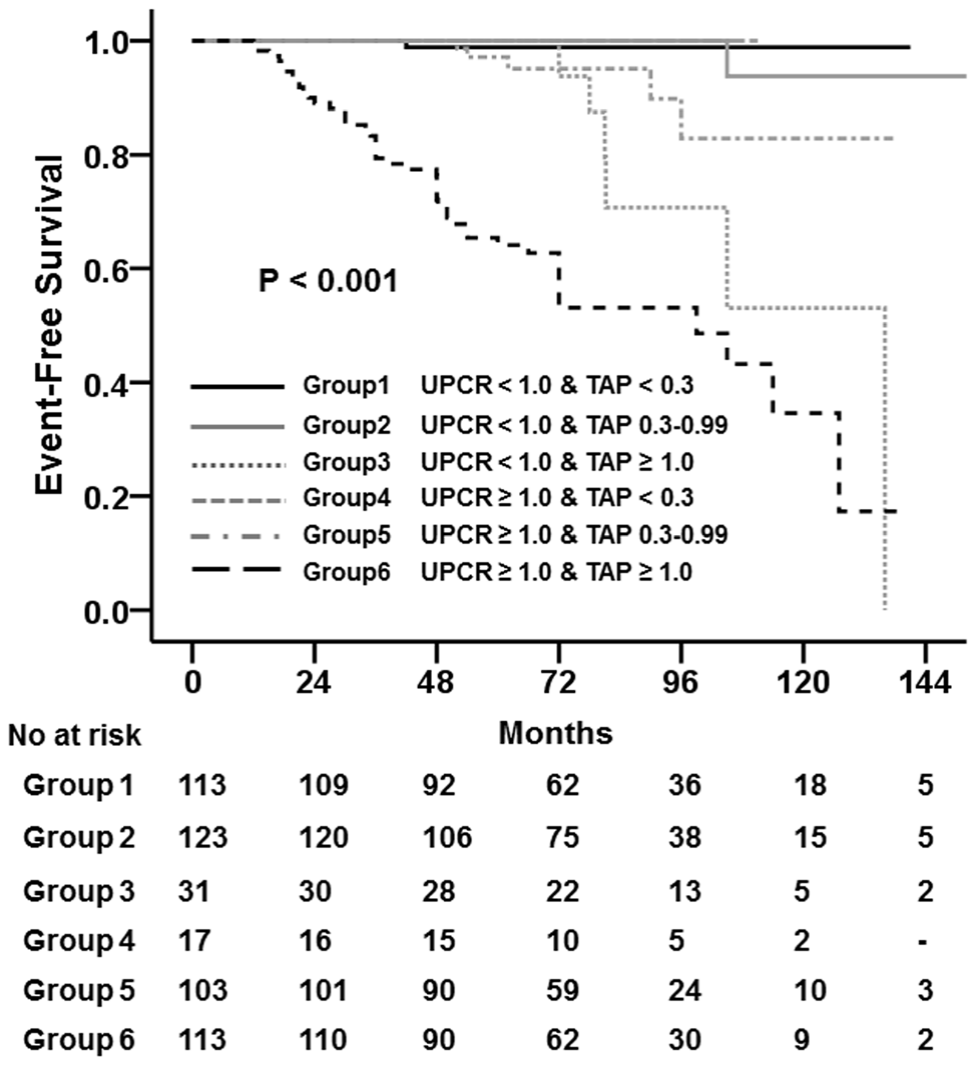
The Kaplan–Meier renal survival curve of patients with IgAN according to baseline urine protein-to-creatinine ratio (UPCR) and time-averaged proteinuria (TA-P). The 10-year renal survival rates were comparable between patients with TA-P of <0.3 g/g and those with TA-P of 0.3–0.99 g/g irrespective of their baseline UPCR. However, if patients had TA-P≥1 g/g during follow-up, their 10-year survival rates were significantly decreased even if they had a baseline UPCR<1.0 g/g.

We further evaluated renal outcomes of patients categorized according to initial proteinuria and TA-P (Table 5). Among 267 patients with baseline UPCR<1.0 g/g, 113, 123, and 31 patients had TA-P of <0.3, 0.3–0.99, and ≥1.0 g/g, respectively. There was no significant difference in the development of a 50% decline in eGFR between patients with TA-P<0.3 g/g and those with TA-P of 0.3–0.99 g/g. However, compared to these two groups, 6 (19.4%) patients with TA-P>1.0 g/g reached a 50% decline in eGFR (*P*<0.001). In addition, among 233 patients with baseline UPCR≥1.0 g/g, 17, 103, and 113 patients had TA-P of <0.3, 0.3–0.99, and ≥1.0 g/g, respectively. There were no patients with TA-P of <0.3 g/g who reached a 50% decline in eGFR. In addition, only 5 (4.9%) patients among the group with TA-P of 0.3–0.99 g/g reached this endpoint. However, 47 (41.6%) patients with TA-P≥1.0 g/g reached a 50% decline in eGFR (*P*<0.001). A Kaplan-Meier curve also showed that the 10-year renal survival rates were comparable between patients with TA-P of <0.3 g/g and those with TA-P of 0.3–0.99 g/g irrespective of their baseline UPCR. However, if patients had TA-P≥1.0 g/g during follow-up, their 10-year survival rates were significantly decreased even if they had a baseline UPCR<1.0 g/g (Figure 3).

**Table 5:** Proteinuria reduction and renal outcome.

			Baseline UPCR<1.0 g/g				Baseline UPCR≥1.0 g/g			
				TA-P (g/g)				TA-P (g/g)		
			<0.3	0.3–0.99	≥1.0	*P*-value	<0.3	0.3–0.99	≥1.0	*P*-value
All										
	N	500	113	123	31		17	103	113	
	↓eGFR>50%	60 (12.0%)	1 (0.9%)	1 (0.8%)	6 (19.4%)	<0.001	0 (0%)	5 (4.9%)	47 (41.6%)	<0.001
	ESRD	34 (6.8%)	0 (0%)	0 (0%)	1 (3.2%)	0.02	0 (0%)	0 (0%)	33 (29.2%)	<0.001
By ARR										
0	N	184	92	74	18		0	0	0	
	↓eGFR>50%	5 (2.7%)	1 (1.1%)	1 (1.4%)	3 (16.7%)	0.001	0 (0.0%)	0 (0.0%)	0 (0.0%)	-
	ESRD	0 (0.0%)	0 (0.0%)	0 (0.0%)	0 (0.0%)	-	0 (0.0%)	0 (0.0%)	0 (0.0%)	-
1	N	149	18	41	9		7	44	30	
	↓eGFR>50%	7 (4.7%)	0 (0.0%)	0 (0.0%)	2 (22.2%)	0.001	0 (0.0%)	1 (2.3%)	4 (13.3%)	0.09
	ESRD	2 (1.3%)	0 (0.0%)	0 (0.0%)	0 (0.0%)	-	0 (0.0%)	0 (0.0%)	2 (6.7%)	0.15
2	N	132	3	8	4		10	45	62	
	↓eGFR>50%	34 (25.8%)	0 (0.0%)	0 (0.0%)	1 (25.0%)	0.229	0 (0.0%)	2 (4.4%)	31 (50.0%)	<0.001
	ESRD	24 (18.2%)	0 (0.0%)	0 (0.0%)	1 (25.0%)	0.229	0 (0.0%)	0 (0.0%)	23 (37.1%)	<0.001
3	N	35	0	0	0		0	14	21	
	↓eGFR>50%	14 (40.0%)	0 (0.0%)	0 (0.0%)	0 (0.0%)	-	0 (0.0%)	2 (14.3%)	12 (57.1%)	0.01
	ESRD	8 (22.9%)	0 (0.0%)	0 (0.0%)	0 (0.0%)	-	0 (0.0%)	0 (0.0%)	8 (38.1%)	0.04

Abbreviations: UPCR, urine protein-to-creatinine ratio; TA-P, time-averaged proteinuria; ARR, absolute renal risk.

Furthermore, the effect of proteinuria reduction was consistently seen across the ARR scores (Table 5). Interestingly, among patients with baseline UPCR<1.0 g/g and ARR = 0, 3 (16.7%) patients who achieved TA-P≥1.0 g/g reached a 50% decline in eGFR. Conversely, a 50% decline in eGFR occurred in only 2 (1.2%) patients with TA-P<1.0 g/g (*P* = 0.001). In addition, among patients who had baseline UPCR≥1.0 g/g and ARR score ≥2, but failed to achieve TA-P<1.0 g/g, a 50% decline in eGFR occurred in 31 (48.4%) and 13 (61.9%) patients with ARR = 2 and 3, respectively. In contrast, only 2 patients from each group with ARR = 2 (3.6%; *P*<0.001) and ARR = 3 (14.3%; *P* = 0.039) reached a 50% decline in eGFR if a TA-P<1.0 g/g was attained.

### Slopes of eGFR among the four TA-P groups

We also compared rates of renal function decline between the four TA-P groups. The eGFR slope was the lowest in patients with TA-P<0.3 g/g, which was significantly lower than in patients with TA-P of 0.3–0.99 g/g (−0.41±1.68 vs. −0.73±2.82 ml/min/1.73 m^2^/year, *P* = 0.03). Moreover, renal function of patients with TA-P of 1.0–2.99 g/g and ≥3.0 g/g deteriorated faster at rates of −4.07±6.54 and −9.35±13.1 ml/min/1.73 m^2^/year, respectively (*P*<0.001) (Table 6).

**Table 6:** The rate of kidney function decline based on 4 categories of time-averaged proteinuria.

		*P* for difference between groups			
TA-P (g/g)	Slope of eGFR decline (ml/min/1.73 m^2^/year)	<0.3	0.3–0.99	1.0–2.99	≥3.0
<0.3	−0.41±1.68	-			
0.3–0.99	−0.73±2.82	0.03	-		
1.0–2.99	−4.07±6.54	<0.001	<0.001	-	
≥3.0	−9.35±13.1	<0.001	<0.001	<0.001	-

Abbreviations: eGFR, estimated glomerular filtration rate; TA-P, time-averaged proteinuria.

## Discussion

This study aimed to determine the optimal target of proteinuria reduction in patients with IgAN. We showed that patients with TA-P<1.0 g/g had favorable outcomes and risk of progression was comparable between patients with TA-P<0.3 g/g and those with TA-P of 0.3–0.99 g/g. However, the rate of kidney function decline was significantly greater in patients with TA-P of 0.3–0.99 g/g than in patients with TA-P<0.3 g/g. These findings suggest that proteinuria reduction to <1.0 g/day suggested by the KDIGO guideline [18] is acceptable, but the ultimate therapeutic goal of proteinuria reduction for renoprotection in IgAN can be modified.

Proteinuria has long been not only a marker of kidney damage but a therapeutic target in the management of various kidney diseases. In diabetic nephropathy, albuminuria is the first clinical sign of diabetic kidney injury. Many studies have consistently shown that improved renal outcome is concordant with the reduction in proteinuria achieved using RAS blockers in patients with overt diabetic nephropathy [19]–[21]. A similar effect of reducing proteinuria is also evident in non-diabetic kidney diseases [22], [23]. Not surprisingly, proteinuria is considered a modifiable risk factor for kidney disease progression. However, excluding some glomerulonephritides which are well responsive to immunosuppressive drugs or undergo spontaneous remission, it is difficult to achieve complete resolution of proteinuria. Moreover, the optimal target for proteinuria reduction to halt progression of kidney disease is unknown.

To date, it has been suggested that proteinuria should be decreased to <1.0 g/day in IgAN because risk of progression increases markedly beyond this point [18], [24], [25]. Using the Toronto Glomerulonephritis Registry, Reich et al. reported that renal survival was similar in patients with TA-P<0.3 g/day and those with TA-P of 0.3–1.0 g/day [11]. A recent Spanish study of 141 IgAN patients with minor abnormalities at presentation also supported this finding because TA-P>0.5 g/day was not associated with an increased risk of developing a >50% increase in baseline serum creatinine levels in the adjusted multivariable analysis compared to TA-P≤0.5 g/day [26]. In keeping with findings of these studies, we showed that there was no difference in renal events between patients with TA-P<0.3 g/g and those with TA-P of 0.3–0.99 g/g. Only 7 (2.0%) patients with TA-P<1.0 g/g developed a 50% decline in eGFR and none of them reached ESRD during follow-up. Their 10-year survival rates were excellent. Furthermore, risk of reaching a 50% decline in eGFR was comparable between patients with baseline proteinuria ≥1.0 g/g who achieved TA-P<1.0 g/g and patients with TA-P<1.0 g/g throughout the follow-up period. These findings together suggest that the cut-off level of proteinuria reduction suggested by the current guideline is reasonable.

Moreover, these findings were consistently observed across all risk groups. We evaluated risk of progression using the ARR. As seen in table 5, irrespective of the ARR score, more patients who failed to achieve TA-P<1.0 g/g reached primary endpoint compared to those who achieved this goal. Interestingly, the effect of proteinuria reduction appears to exist even in high risk patients. There were only 4 patients with TA-P<1.0 g/g who developed a 50% decline in eGFR although they had baseline proteinuria ≥1.0 g/g and ARR≥2. These findings further underscore the importance of proteinuria reduction <1.0 g/g in the management of IgAN.

On the other hand, there has been emerging concern about whether further reduction of proteinuria below the level the current guideline suggests is required. In a Chinese cohort study by Le et al. [12], TA-P of 0.5–1.0 g/day were associated with a 9.1-fold increased risk of progression compared to TA-P<0.5 g/day. In addition, rate of eGFR decline was greater in patients with TA-P of 0.5–1.0 g/day. Unfortunately, tubulointerstitial fibrosis and glomerulosclerosis, which are widely accepted as the most important pathologic factors affecting renal outcome, were not evaluated in their study. In contrast, the Spanish cohort study and the present study incorporated pathologic findings in the multivariable analyses and these two studies did not find a significant risk reduction of developing adverse renal events conferred by further lowering proteinuria among patients with TA-P<1.0 g/day or 1.0 g/g. However, in our study, there were slightly more patients with TA-P of 0.3–0.99 g/g who developed a 50% decline in eGFR than those with TA-P<0.3 g/g (2.7% vs. 0.8%, *P* = NS) and the former group had a trend towards a higher risk of reaching the primary outcome although these did not reach statistical significance. In addition, from a viewpoint of eGFR slope, our findings are in agreement with the Chinese cohort study. In fact, the Oxford MEST score was higher in patients with TA-P of 0.3–0.99 g/g than in those with TA-P<0.3 g/g. This higher frequency of pathologic lesions in the former group might result in a greater slope of eGFR decline than in the latter group, although the annual eGFR decline was −0.73 ml/min/1.73 m^2^ in patients with TA-P of 0.3–0.99 g/g, which is considered acceptable in management of patients with chronic kidney disease. However, given the slowly progressive nature of IgAN and young age at the time of diagnosis, this difference in eGFR slope between patients with TA-P of 0.3–0.99 g/g and those with TA-P<0.3 g/g may lead to difference in long-term prognosis. Of note, in both Chinese cohort study and our study, patients with minimal TA-P rarely progress; their eGFR slopes were only −0.4 ml/min/1.73 m^2^/year. These findings suggest a potential renoprotective benefit of further lowering proteinuria <0.3 g/g or <0.5 g/day to halt progression of IgAN.

The present study also partly supports the previous findings that the target for proteinuria reduction differs depending on the type of proteinuric glomerular diseases [14], [27]. In other glomerulonephritides such as membranous nephropathy (MGN) or focal segmental glomerulosclerosis, patients with proteinuria of 1.0–2.0 g/day had similar eGFR decline rate to those with proteinuria of 0.5–1.0 g/day [14]. In particular, the rate of decline of eGFR in patients with MGN who had proteinuria <3.5 g/day was only −0.93 ml/min/1.73 m^2^/year and 12.0% of these patients developed a 50% decrease in creatinine clearance during a median follow-up of 82 months [27]. In contrast, in the Toronto study by Reich et al, renal survival sharply began to decline as TA-P increased from 1.0–2.0 g/day in patients with IgAN [11]. This finding was corroborated by our study showing that the risk of reaching a 50% decline in eGFR began to increase markedly as TA-P rose above 1.0 g/g. The 10-year renal survival rate of patients with TA-P<1.0 g/g was >90% and none of these patients developed ESRD, whereas that of patients with TA-P of 1.0–2.99 g/g was 47.7% and 20.0% developed ESRD. Therefore, unlike other types of glomerulonephritis in which subnephrotic proteinuria is not associated with higher risk of loss of kidney function, aggressive treatment to lower proteinuria to 1.0 g/g can be justified in patients with IgAN.

Several shortcomings of this study should be discussed. First, effects of therapeutic interventions such as RAS blockers or corticosteroids could not be accurately assessed due to retrospective nature of our study. The KDIGO guideline suggests the use of corticosteroids in patients with persistent proteinuria >1.0 g/day and preserved kidney function [18], [28], [29]. In this study, corticosteroids were prescribed in 56 (11.2%) patients. Of note, 6 (4.6%) and 25 (11.1%) patients with TA-P<0.3 g/g and 0.3–0.99 g/g received corticosteroids, respectively. 23 (74.2%) of these patients initially had persistent proteinuria >1.0 g/g, despite optimized supportive care for at least 3 months and the remaining 8 (25.8%) showed nephrotic syndrome at presentation. All these patients responded to corticosteroids and achieved a favorable outcome. Although this finding may favor the use of corticosteroids, it was not associated with an improved renal outcome in the fully adjusted multivariable model. More well-designed prospective studies are required to validate the effect of corticosteroids. Second, we used UPCR for the measurement of proteinuria instead of 24-h urine collection, the gold standard method to assess proteinuria. In fact, there was a significant difference between baseline 24-h proteinuria and UPCR as seen in table 1. Unfortunately, 24-h urine collection was not feasible at all visits and is usually repeated only when proteinuria substantially increases. Because UPCR is easily performed in the outpatient setting and can be reliably used to monitor proteinuria [30], our institution performs UPCR at every visit instead of 24-h urine collection. Therefore, in this study, calculating TA-P based on UPCR more accurately reflected the average proteinuria during the follow-up period. Finally, our median follow-up duration was 65 months, which is shorter than in previous studies [11], [26]. During this period, ESRD, a ‘hard’ endpoint, did not occur in patients with proteinuria <1.0 g/g and thus it is not feasible to evaluate whether this outcome may differ between patients with TA-P<0.3 g/g and those with TA-P of 0.3–0.99 g/g. In addition, only 7 patients with TA-P<1.0 g/g reached a 50% decline in eGFR. Such small number of events might not have adequate statistical power to see the difference in the study endpoints. As aforementioned, patients with TA-P of 0.3–0.99 g/g had a higher Oxford-MEST score and a greater decline rate of renal function than patients with TA-P<0.3 g/g although eGFR slope in the former group was >−1.0 ml/min/1.73 m^2^/year. Because IgAN has slowly progressive nature, a longer period of observation is required to evaluate whether more renal events will occur in patients with moderate proteinuria.

## Conclusions

This study showed that the risk of developing adverse renal events was comparable between patients with TA-P<0.3 g/g and those with TA-P of 0.3–0.99 g/g. In addition, irrespective of initial proteinuria, their renal outcome was favorable if they achieved TA-P<1.0 g/g. However, patients with TA-P of 0.3–0.99 g/g had a greater slope of eGFR decline than those with TA-P<0.3 g/g. These findings suggest that primary therapeutic target of proteinuria reduction is <1.0 g/g, but the ultimate optimal goal can be lowered to <0.3 g/g to halt progression of IgAN. Further long-term studies are required to clarify whether proteinuria reduction to <0.3 g/g may confer better renal outcomes.
